# The prognostic value of postoperative radiotherapy in right tumor for lung related death: based on SEER database and real-world data

**DOI:** 10.3389/fonc.2023.1178064

**Published:** 2023-04-06

**Authors:** You Mo, Minxin Chen, Minglei Wang, Meng Wu, Jinming Yu

**Affiliations:** ^1^ The First Affiliated Hospital of Shantou University Medical College, Shantou, Guangdong, China; ^2^ Department of Oncology, The Affiliated Hospital of Southwest Medical University, Luzhou, Sichuan, China; ^3^ Department of Radiation Oncology, Shandong Cancer Hospital and Institute, Shandong First Medical University and Shandong Academy of Medical Sciences, Jinan, Shandong, China

**Keywords:** IIIA-N2, non-small-cell lung cancer, postoperative radiotherapy, overall survival, lung laterality

## Abstract

**Background:**

Postoperative radiotherapy (PORT) is a therapeutic strategy for patients with non-small cell lung cancer (NSCLC). Nevertheless, some studies suggesting PORT does not improve overall survival (OS) including Lung ART phase III trial. The role of PORT and high-risk groups need to be confirmed.

**Methods:**

Patients from the Surveillance, Epidemiology, and End Results program (SEER) from 2004 to 2015 were eligible. Aged ≥18 years with stage IIIA-N2 NSCLC, accepted PORT or not were considered for the study. Cox regression analyses and multivariate competing risk model were performed. Propensity score matching (PSM) was conducted. Data from a single-center study in China were used for validation.

**Results:**

In all patients with IIIA-N2 NSCLC, death from respiratory illness increased year by year, with right lung-related deaths accounting for the main proportion. In SEER database, PORT was detrimental for OS after PSM (hazard ratio [HR], 1.088; 95% CI, 1.088–1.174; *P* = 0.031), with a same trend for death from the lungs (HR, 1.13; 95% CI, 1.04–1.22; *P* = 0.005). Right tumor receiving PORT were prone to death from lung disease(HR, 1.14; 95% CI, 1.02–1.27; *P* = 0.018). In China single-center cohort, PORT was significantly correlated with deteriorated OS (HR 1.356; 95% CI 1.127–1.632; *P <*0.01), especially in the right laterality (HR 1.365; 95% CI 1.062–1.755; *P* = 0.015).

**Conclusions:**

PORT was a risk factor for stage IIIA-N2 NSCLC patients, particularly with characters of right laterality, male sex, age ≥65 years, and advanced tumor stage. These patients are more likely to death from lung disease after PORT.

## Introduction

Non-small-cell lung cancer (NSCLC) accounts for more than 80% of all lung cancers (1). Although there are various treatment modalities, surgery is a radical treatment for patients with IIIA NSCLC, with only a small percentage of patients treated surgically. This is because most patients are diagnosed with locally advanced or metastatic disease, and patients who receive surgery alone are prone to subsequent local recurrence and distant metastasis. Therefore, multidisciplinary sequential therapies are considered the optimal treatment regimen (2). For patients with operable stage IIIA-N2 NSCLC, treatments recommended in AJCC are surgery plus PORT or concurrent chemoradiotherapy (3). The combination treatment standard is widely used, and previous studies have repeatedly proven that PORT, as a part of standard treatments, can prevent local recurrence. However, it is still inconclusive whether PORT can improve OS and prevent serious adverse effects even death in patients with IIIA-N2. In a single-center, randomized, controlled study published in JAMA in 2021, 364 patients with IIIAN2 were divided into control and postoperative radiotherapy groups. Results suggest that PORT did not improve median DFS(hazard ratio [HR], 0.84; 95% CI, 0.65-1.09; *P* = 0.20) (4). In the 2020 ESMO conference: The Lung ART study showed that PORT had similar results in DFS (3-year: 47.1% *vs*. 43.8%; *P* = 0.16) or OS (3-year: 66.5% *vs*. 68.5%; HR, not provided yet) in patients with pIIIA-N2 NSCLC. Not only that, the PORT group had a higher rate of grade 3-4 toxicities and more deaths from cardiopulmonary complications (2.0% *vs* 16.2%) (5).

Even though there were positive conclusions in other studies. For example, a study of the National Cancer Database which identified 4,483 patients with N2 NSCLC after complete resection and adjuvant chemotherapy. PORT was associated with an increase in median (45.2 *vs*. 40.7 months) and 5-year OS (39.3% *vs*. 34.8%, *P =* 0.014) (6). Another research based on a prospectively maintained nationwide oncology outcomes database demonstrated PORT had a similar effect (HR = 0.81; 95% CI: 0.70-0.94; *P* = 0.005) (7).

Generally, due to subjective observed variables and inclusion criteria, there was no consistent conclusion. Therefore, we investigated the association between PORT and survival and categorize the survival outcomes by the cause of death in different regions with the SEER database. We hope to demonstrate who are at high risk after PORT for patients with IIIA NSCLC and provide guidance for those who will actually benefit.

## Materials and methods

### Patient selection

A total of 579,723 patients were screened for our current study (patient population from 2004 to 2015), retrospectively assessed from the SEER database through searching for lung and bronchus as the keywords firstly.

Among the patients screened, 123,927 were excluded because they did not fit the pathological characteristics where ICD grade 0–3 or behavior of NSCLC was required. After a new round of screening, we identified a population of 39,147 patients potentially eligible for analysis who were diagnosed with stage IIIA-N2 NSCLC. Next, 11,778 patients were excluded because they were <18 years old, had a survival time of less than 1 month, or this was not their primary tumor. Furthermore, the patients were included whose treatments were surgery and chemotherapy, with or without PORT. Thus, a population of 5,741 patients was deemed eligible for the study based on our strict criteria. Finally, we divided the population into left and right lungs based on their tumor location and carried out PSM separately.

An independent cohort of 793 patients with stage IIIA-N2 NSCLC from Shandong Cancer Hospital in China was used for external validation. These patients met the same inclusion and exclusion criteria as those from the SEER Database.

### Ethics statement

This study was mainly based on the SEER database and was conducted in compliance with the Declaration of Helsinki. Permission was obtained to access the files of the SEER program research data (reference number 18157-Nov2019). Informed consent was not required because the patients were not personally identified. This study was approved by the Ethics Review Committee of Shandong Cancer Hospital, affiliated with Shandong University.

### Statistical analysis

The statistical analyses were performed using IBM SPSS Statistics version 25.0 software (IBM Corp). StataMP 16 and Graph Pad Prism 8.0 (GraphPad Software) were used for cartography. The variables analyzed for all patients included race, sex, age, tumor grade, laterality, tumor histology, and staging. The observation endpoints of the study were overall survival (OS), death from respiratory illness, death from cardiac disease, and other causes of death extracted from the SEER database. Baseline characteristics of different groups were compared using chi-square tests. Propensity score matching (PSM) analysis was used to minimize latent differences by matching patient characteristics. Multivariate competing risk analysis was used to calculate lung-specific death. We evaluated the risk of mortality and conducted subgroup analyses using univariate and multivariate Cox proportional hazards regression models. All statistical tests were two-sided, and statistical significance was set at *P <*0.05.

## Results

### Temporal trends of different causes of death

**Figure 1:**
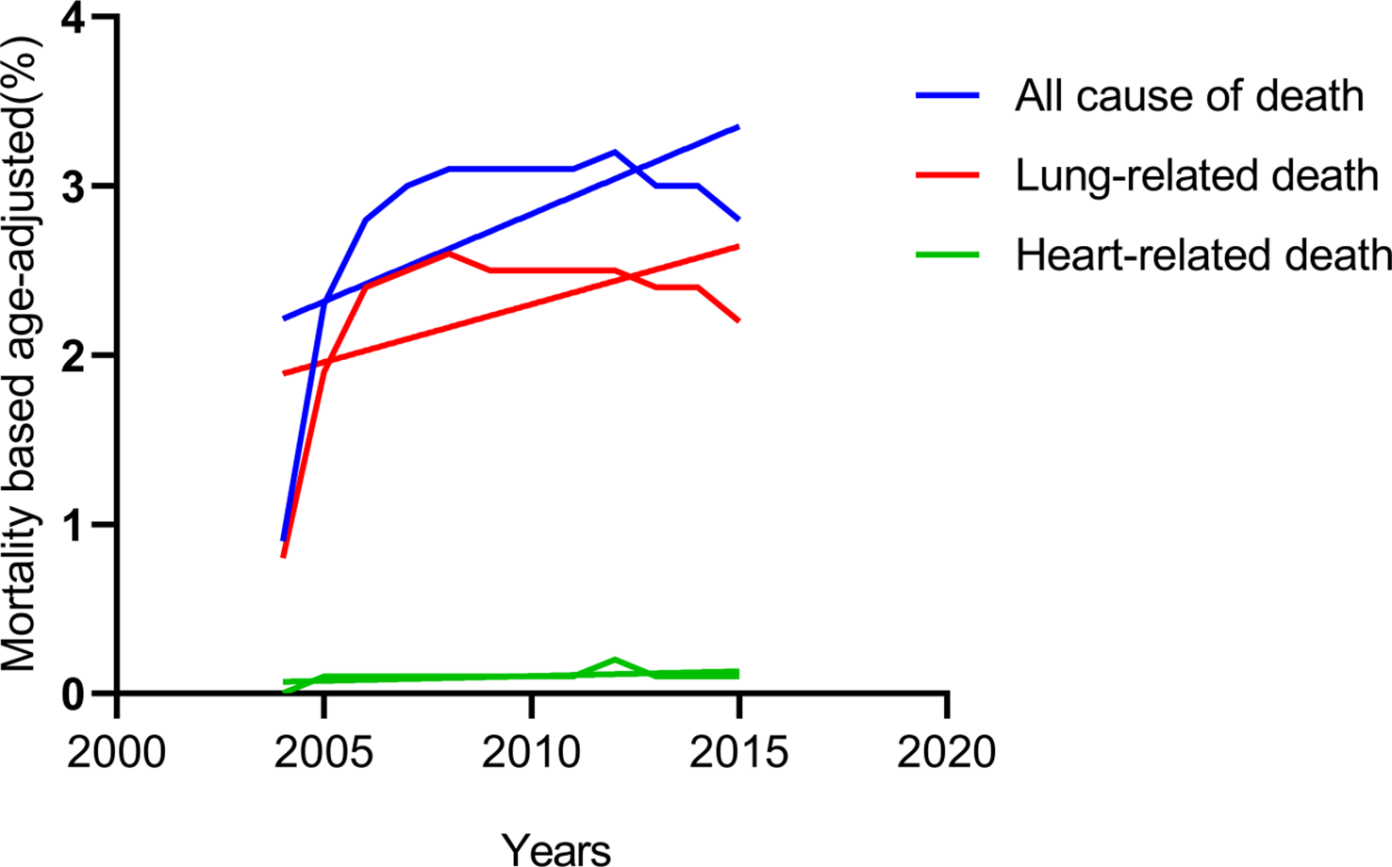
Temporal trends of different causes of death.

**Figure 2:**
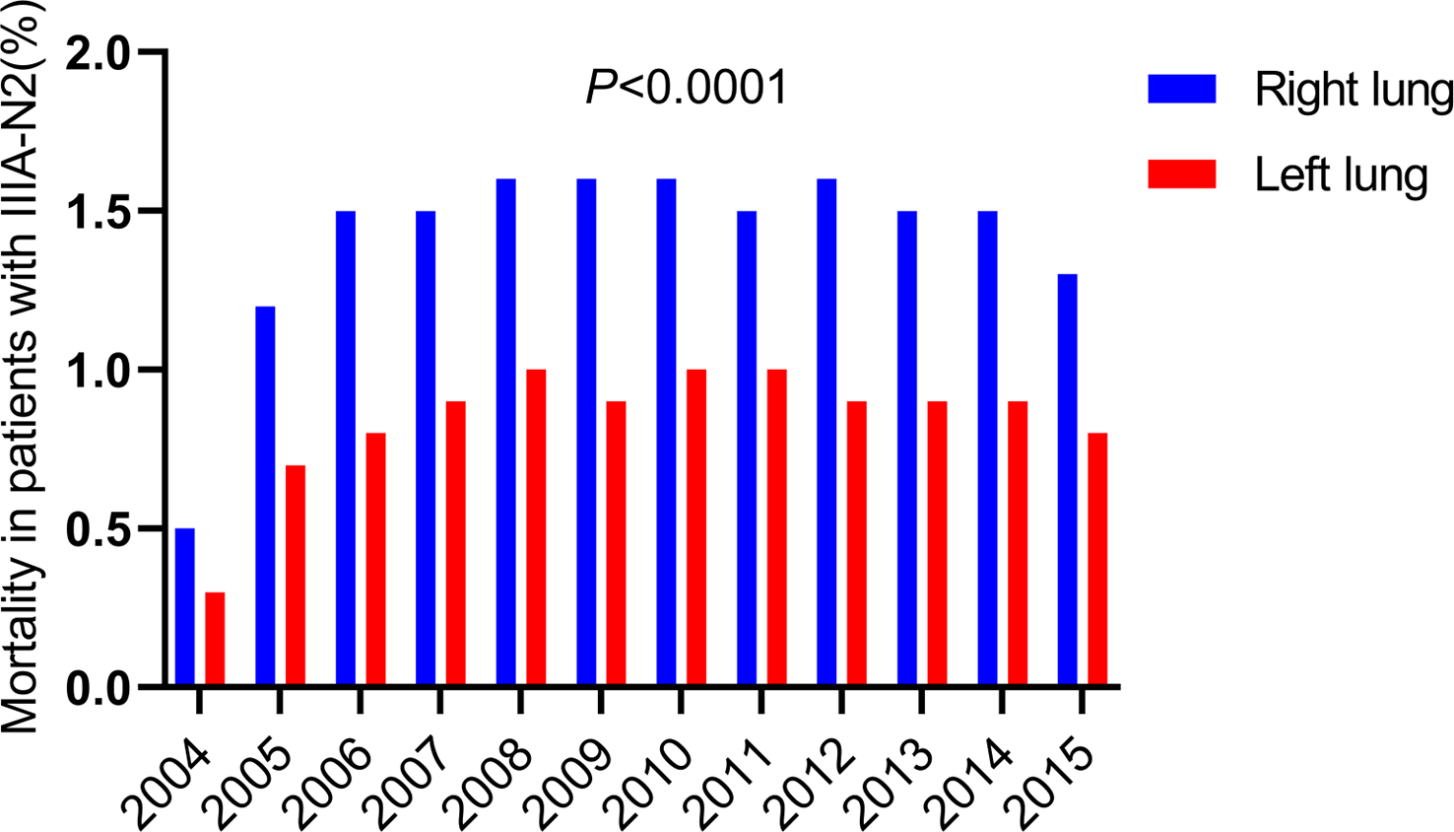
Death of different laterality in death from the lungs with IIIA-N2 stage NSCLC in 2004-2015.

By classifying the causes of death in all patients with IIIA-N2 NSCLC from 2004 to 2015, we found that heart-related deaths that remained low all the time, while lung-related deaths increased over time and maintained the same trend as all causes of death **(** **Figure 1** **)**. Considering of the anatomy of the lungs, we spontaneously separate the population into left and right lateralities for subgroup analysis. The results showed that in patients with IIIA-N2 NSCLC, the death rate from the right lung was significantly higher than that from the left lung (*P <*0.001) as **Figure 2**.

### Patient demographics

**Figure 3:**
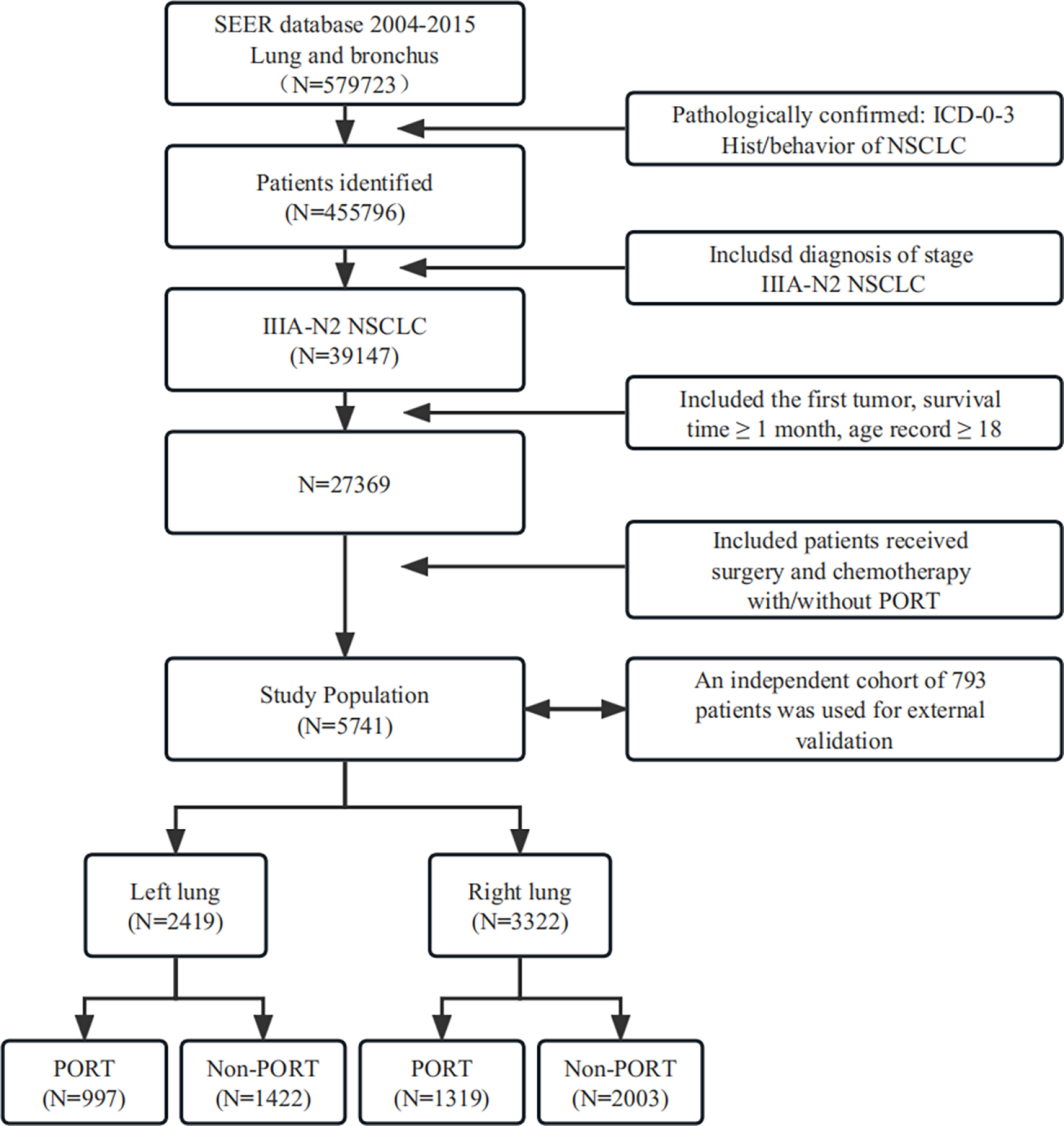
Selection strategy of patients.

A total of 5,741 patients were included in our retrospective cohort study. By dividing by laterality, 2419 patients were of left lung laterality and the rest was right lung laterality **(** **Figure 3** **)**. An obvious imbalance was noted in race, staging, tumor histology type, and tumor grade. Considering that a basis might cause an unknown effect, we performed propensity score matching on all enrolled patients and subgroups to remove confounding factors. The entire cohorts of patient characteristics were shown in **Table 1A**. Since none of the subsequent analyses in the left laterality were statistically significant, we showed it in the **Supplementary Table 1**.

**Table 1A:** Baseline Characteristics of Patients with IIIA-N2 NSCLC of OS.

Variables	Radiation (%)	None (%)	*P*
Race			0.436
White	1884 (50.3)	1862 (49.7)	
Black	238 (50.3)	235 (49.7)	
Others	194 (47.0)	219 (53.0)	
Sex			0.702
Female	1143 (49.7)	1156 (50.3)	
Male	1173 (50.3)	1160 (49.7)	
Age,years			0.975
<65	772 (50.0)	773 (50.0)	
≥65	1544 (50.0)	1543 (50.0)	
Grade			0.42
I	976 (49.4)	1000 (50.6)	
II	1086 (51.0)	1044 (49.0)	
III	254 (48.3)	272 (51.7)	
Laterality			0.678
Left	997 (50.4)	983 (49.6)	
Right	1319 (49.7)	1333 (50.3)	
Tumor histology			0.886
squamous and transitional cell	502 (50.2)	498 (49.8)	
Adenocarcinoma	1814 (49.9)	1816 (50.1)	
Others			
Tumor stage			<0.001
I	662 (52.9)	590 (47.1)	
II	1236 (49.1)	1279 (50.9)	
III	400 (47.4)	443 (52.6)	
IV	18 (81.8)	4 (18.2)	

### Univariate and multivariate analysis of OS

In the Cox regression hazard analysis, we examined univariate variables, including race, sex, age, grade, laterality, tumor histology, tumor stage, and PORT. According to the results of univariate Cox regression analysis, race, sex, age, tumor histology, and tumor stage were significantly associated with a shorter OS after PSM (*P <*0.05). In the multivariate analysis, sex, age, and tumor stage had a similar statistical significance (*P <*0.05) and PORT was detrimental to OS (HR, 1.088; 95% CI, 1.088–1.174; *P* = 0.031). The detailed results are shown in **Supplementary Table 2**.

### Subgroup analysis after PSM

**Figure 4:**
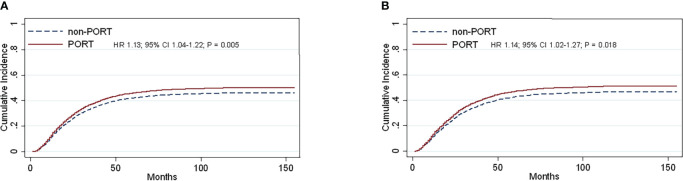
Cumulative incidence estimates of stage IIIA-N2 NSCLC patients by PORT (*vs* non-PORT) after PSM in death from the lungs **(A)** and (PORT.*vs* non-PORT) in death from the lungs in right laterality **(B)**.

Contacting the causes of death analysis of all patients in 2004-2015, we defined death from respiratory illness as the observed outcome and the others as competitive events. Subgroup analysis suggested that several variables, including age (HR, 1.12; 95% CI, 1.02–1.22; *P* = 0.012), sex (HR, 0.84; 95% CI, 0.77–0.91; *P <*0.0001), tumor stage (HR, 1.22; 95% CI, 1.15–1.30; *P <*0.0001) **(** **Supplementary Figure 1**), and PORT (HR, 1.13; 95% CI, 1.04–1.22; *P* = 0.005) were confirmed to be convincing prognostic markers for lung-specific death **(** **Figure 4A** **)**.

### Analysis of different laterality after PSM

Multivariate competing risk analysis was performed repeatedly for the subgroup analysis. No statistically significant differences were found in left laterality until the right laterality analysis was conducted. After PSM was completed, the total number of patients was 2,638, and the baseline balance was maintained between subgroups. The statistically different variables, including age (HR, 1.20; 95% CI, 1.07–1.35; *P* = 0.002), sex (HR, 0.82; 95% CI, 0.73–0.91; *P <*0.0001), and tumor stage (HR, 1.20; 95% CI, 1.11–1.29; *P <*0.0001) (**Supplementary Figure 1**) showed their respective effects on lung-specific death. In particular, PORT was prone to death from the lung (HR, 1.14; 95% CI, 1.02–1.27; *P* = 0.018) **(** **Figure 4B** **).** The entire cohorts of patient characteristics in right laterality were shown in **Table 1B**.

**Table 1B:** Baseline Characteristics of Patients with IIIA-N2 NSCLC in right laterality of OS.

Variables	PORT (%)	Non-PORT (%)	*P*
Race			0.615
White	1061 (50.1)	1055 (49.9)	
Black	137 (47.6)	151 (52.4)	
Others	121 (51.7)	113 (48.3)	
Sex			0.876
Female	650 (49.8)	654 (50.2)	
Male	669 (50.1)	665 (49.9)	
Age,years			0.219
<65	439 (48.3)	469 (51.7)	
≥65	880 (50.9)	850 (49.1)	
Grade			0.225
I	547 (49.1)	566 (50.9)	
II	614 (51.7)	574 (48.3)	
III	158 (46.9)	179 (53.1)	
Tumor histology			0.923
squamous and transitional cell	268 (50.2)	266 (49.8)	
Adenocarcinoma	1051 (50.0)	1052 (50.0)	
Others			
Tumor stage			0.632
I	371 (50.4)	365 (49.6)	
II	698 (50.3)	689 (49.7)	
III	239 (48.1)	258 (51.9)	
IV	11 (61.1)	7 (38.9)	

### Validation of the effect of PORT in the single-center cohorts

**Figure 5:**
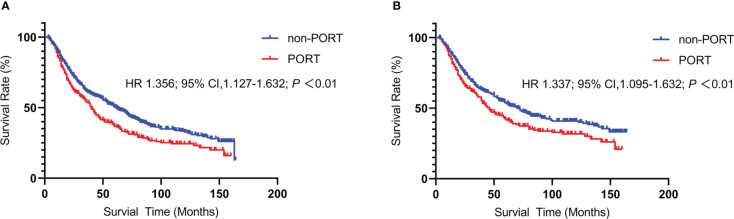
Kaplan-Meier curve of Overall Survival and lung-related death between the PORT group and the non-PORT group: Overall Survival **(A)** and lung-related death **(B)**.

A total of 793 patients in the single-center cohort were included. Similar to the above findings, PORT was significantly correlated with deteriorated OS compared to non-PORT group (mOS, 40 *vs* 62months, HR 1.356; 95% CI 1.127-1.632; *P*<0.01) **(** **Figure 5A** **)** and lung-related death (46 *vs* 71months, HR 1.337; 95% CI 1.095–1.632; *P*<0.01) **(** **Figure 5B** **)**. Impact of PORT on death from lung cancer was further evaluated among different clinically defined subgroups (according to age at diagnosis, sex, race, grade, primary site, and stage). Results have been shown in the **Supplementary Figure 2**. The result showed that age ≥60 years (HR 1.433; 95% CI, 1.135-1.808; *P* = 0.002), tumor stage T2 (HR 1.438; 95% CI, 1.106-1.869; *P* = 0.007), T3 (HR 2.293; 95% CI, 1.445-3.369; *P* = 0.000) and PORT for right lung (HR 1.365; 95% CI 1.062–1.755; *P =* 0.015) have higher risk and may be detrimental to patients.

## Discussion

For some patients who were amenable to surgery with IIIA-N2 NSCLC, high risks of recurrence and death rates were still troublesome even after complete resection (8). IIIA-N2 stage NSCLC was highly heterogeneous, and multidisciplinary treatments were gradually being used to overcome them. For instance, postoperative chemotherapy (POCT) was recommended in the NCCN Guidelines as a standard treatment to help control local recurrence and prolong OS (9). Similarly, PORT could destroy residual tumors and local subclinical lesions as a local treatment, thus theoretically reducing the recurrence rate and improving the therapeutic effect. However, disagreements regarding the efficacy of PORT had existed. The postoperative pathological stage determined whether PORT was an essential treatment. It was unclear whether patients could benefit from PORT at stage IIIA-N2. Considering the influence that the RTOG 0617 trial and Lung ART would have progressed, it was imperative to evaluate different causes of death after PORT using historical data. Therefore, we conducted this research to verify and analyze the efficacy of PORT and to identify high-risk patients.

### PORT is significant to OS

Cox regression analysis and the competitive risk model implied the significant adverse effect of the male, age ≥60 years, advanced tumor grade and PORT for OS which cause death from the lungs similarly. The detrimental effect of the first four factors on OS had been a consensus in previous studies. However, the role of PORT remained unknown. An earlier study showed that PORT had a detrimental effect on survival in the early stage IIIA (HR, 1.21; 95% CI, 1.08–1.34), whereas there was no clear evidence of its adverse effects on IIIA-N2 NSCLC (10). In addition, there were positive opinions regarding PORT. In some analyses, PORT was believed to result in a significantly lower local-regional relapse and improved OS (11). Similarly, in another remarkable study by Lally et al., survival of patients who received PORT with stage IIIA-N2 NSCLC was significantly improved (HR, 0.855; 95% CI, 0.762–0.959; *P* = 0.008) (12). In terms of our research results, a possible cause of the adverse survival effect of PORT was that the damage caused by the operation could weaken the patient’s immunity and tolerance to irradiation. In addition, we noticed that the incidence of radiation pneumonia was reduced and OS decreased due to significant advances in radiotherapy equipment and technology (13). Considering that the diagnosis time of our patients was from 2004–2015, the limitations in technology might have contributed to the worse OS.

### PORT is significant to the lung-specific death

Previous studies had reported that PORT was related to death from heart disease in patients with NSCLC (HR, 1.30; 95% CI, 1.08–1.34; *P* = 0.02) (14). However, our research results suggested that the specific effect of PORT was on the lungs rather than the heart. A possible explanation was that many normal tissues exist in the thorax, which are organs at risk (OARs) and often dose-limiting during thoracic radiotherapy (RT) (15). The most important organ was the heart, so we paid the most attention to the irradiation dose. Mortality from cardiac events was prevented by the increasing use of advanced radiation modalities and the introduction of cardiac radiation dose constraints (14). At the same time, technicians tried to strike a balance between covering the entire target area and reducing out-of-field exposure to avoid the impact of radiation on the heart in clinical practice. However, as the largest surface area organ in the thoracic cavity, we might have ignored the effect of radiation on the lungs.

In addition, dosimetric analysis concluded that a regional dose was associated with survival, whereas a cardiac mean dose, V5, and V30 showed no significance (16). The mean total lung V20, even V20 >12%, remained a significant predictor of radiation pneumonitis or poor OS, even though it was a clinical consequence (17).

These studies indicated that the lungs are more sensitive to radiation than the heart, which might explain the significant difference in lung-specific death rather than cardiac-specific death in our study.

### PORT is significant to the lung-specific death in right laterality

Regarding the special effect of PORT on right laterality in our research, we had the following explanations. Above all, we noticed that cardiotoxicity after PORT for left lung NSCLC tumors had been reported in multiple studies. Although the technology had improved, the cardiotoxicity of chest radiotherapy was inevitable and even increased (14, 18). Another clinical trial pointed out that higher cardiovascular death was associated with women with left breast cancer who received radiotherapy (19). It was revealed that the laterality of cancer seemed to have some special effects on patients’ survival outcomes. Based on these results, we hypothesized that direct irradiation causes serious organ injury and even death. The heart was mainly affected by left chest RT. Therefore, with PORT for right lung tumors, the right lung was completely exposed to irradiation without shielding from the heart. In addition, some studies had suggested that radiotherapy increases the risk of RP when tumors grow in the lower lobes owing to a greater density of target cells (20, 21). Whether it has a greater density in the right lung should be explored in future studies.

Furthermore, we considered that poor survival of the right-sided tumor in the PORT group might be associated with local recurrence. To date, several typical findings had attracted attention. Kelsey et al. (22) illustrated that the location and distribution of local recurrent sites in different pulmonary lobes might be determined by the respective routes of lymphatic drainage. Although left lung cancer was complex, perivascular, paraaortic, and the AP window was frequently involved, the metastatic pathway of right-sided tumors resulted in ipsilateral paratracheal. However, in the POCRT group, the bronchial stump had a higher therapy failure rate. This conclusion was confirmed in a previous study, which suggested that the sites of therapy failure were based on the lobe in which the primary tumor was located. Ipsilateral recurrences were dominant in right-sided tumors, whereas most left-sided tumors frequently involved the contralateral mediastinum in III N2 NSCLC patients who underwent POCT (23). Coincidentally, the study by Billiet et al. showed that it was mainly a bilateral local recurrence pattern in left-sided tumors (24). Consequently, right-sided tumors were at an increased risk of relapse and might not benefit from PORT for local lesions. As we know, PORT CTV of lung cancer on different sides was based on comprehensive surgical, lymph node involvement distribution, and mediastinal lymphatic drainage imaging evidence (25), but we cannot analyze the role of these factors in the right-sided tumor group in this study. Therefore, further studies were needed to verify and analyze the efficacy of PORT and to identify high-risk patients.

There were several limitations to our study. First, the use of PORT was not random but rather influenced by patients’ and physicians’ preferences because it was a retrospective study. Moreover, the SEER database did not provide data regarding disease recurrence, total radiation dose, and fractionation schedule. Thus, we were not able to assess the impact of these factors on OS. Additionally, relevant information about the lymph nodes was not queried. These issues need to be explored in future studies to contribute to the improvement of clinical guidelines.

## Conclusion

PORT is detrimental to patients with stage IIIA-N2 NSCLC, especially those with right laterality, male sex, age ≥65 years, and advanced tumor stage. These conclusions are varified in real-world data. Therefore, PORT should be cautiously applied by clinicians with these characters to improve the OS.

## Data availability statement

The raw data supporting the conclusions of this article will be made available by the authors, without undue reservation.

## Author contributions

All authors contributed to the article and approved the submitted version.

## Supplementary material

Supplementary Figure 1 Cumulative incidence estimates of stage IIIA-N2 NSCLC patients by key characteristics after PSM. The observed event are death from the lungs from **(A–C)** and the observed event are death from the lungs in right laterality from **(D–F)**. Categorical variables including S1A: sex; S1B: age; S1C: tumor stage; S1D: sex; S1E: age; S1F: tumor stage.

Supplementary Figure 2 Forest plots displaying the relationship between the PORT group and the non-PORT within the different subgroups.
