# Supplementary figures and images for: The prognostic value of postoperative radiotherapy in right tumor for lung related death: based on SEER database and real-world data
Source: Front Oncol. 2023 Apr 6;13:1178064. doi: 10.3389/fonc.2023.1178064 (PMC10117832; doi:10.3389/fonc.2023.1178064)

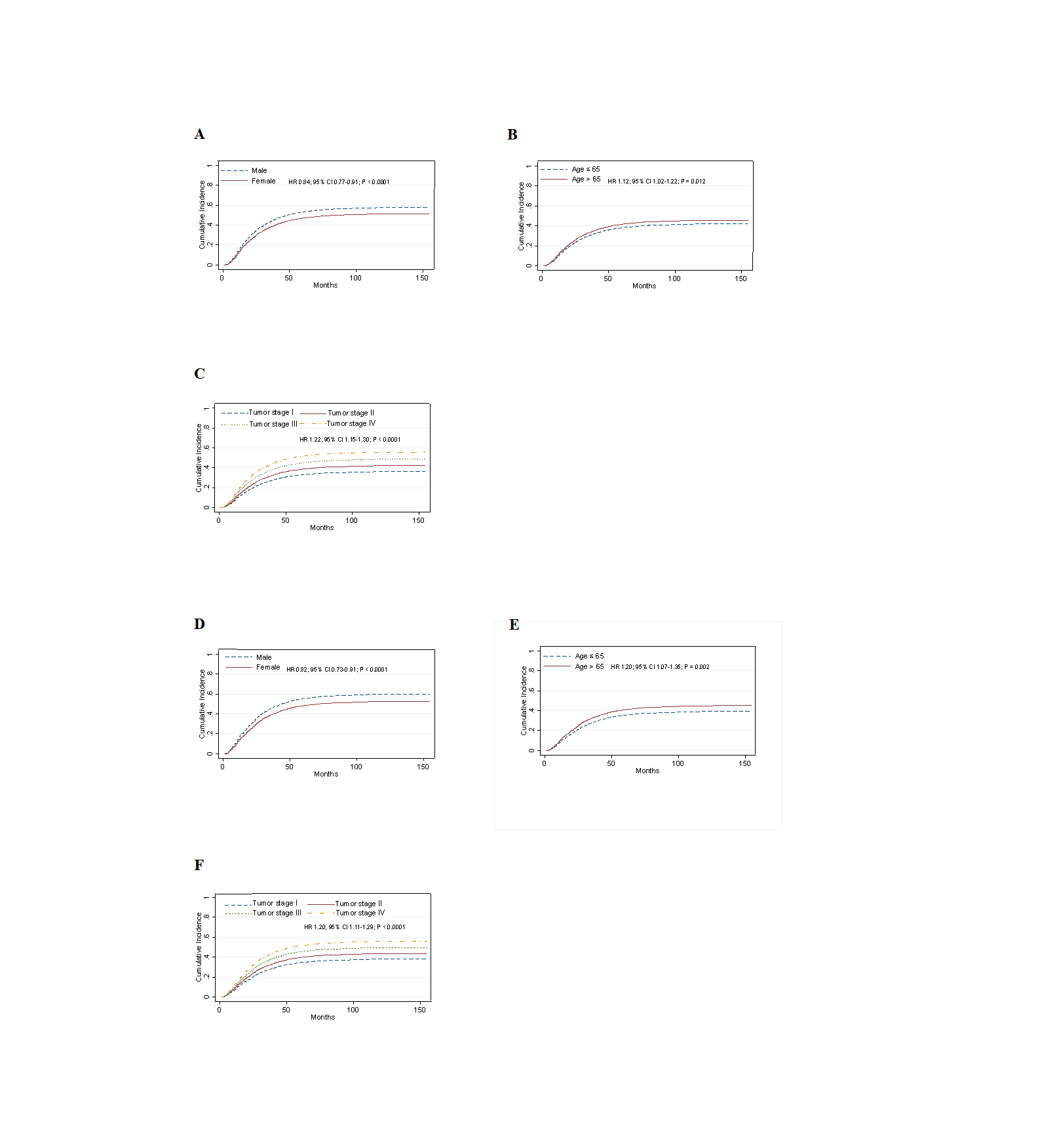

Supplement: Supplementary Figure 1 — Cumulative incidence estimates of stage IIIA-N2 NSCLC patients by key characteristics after PSM. The observed event are death from the lungs from (A–C) and the observed event are death from the lungs in right laterality from (D–F). Categorical variables including S1A: sex; S1B: age; S1C: tumor stage; S1D: sex; S1E: age; S1F: tumor stage. [file Image_1.tif]

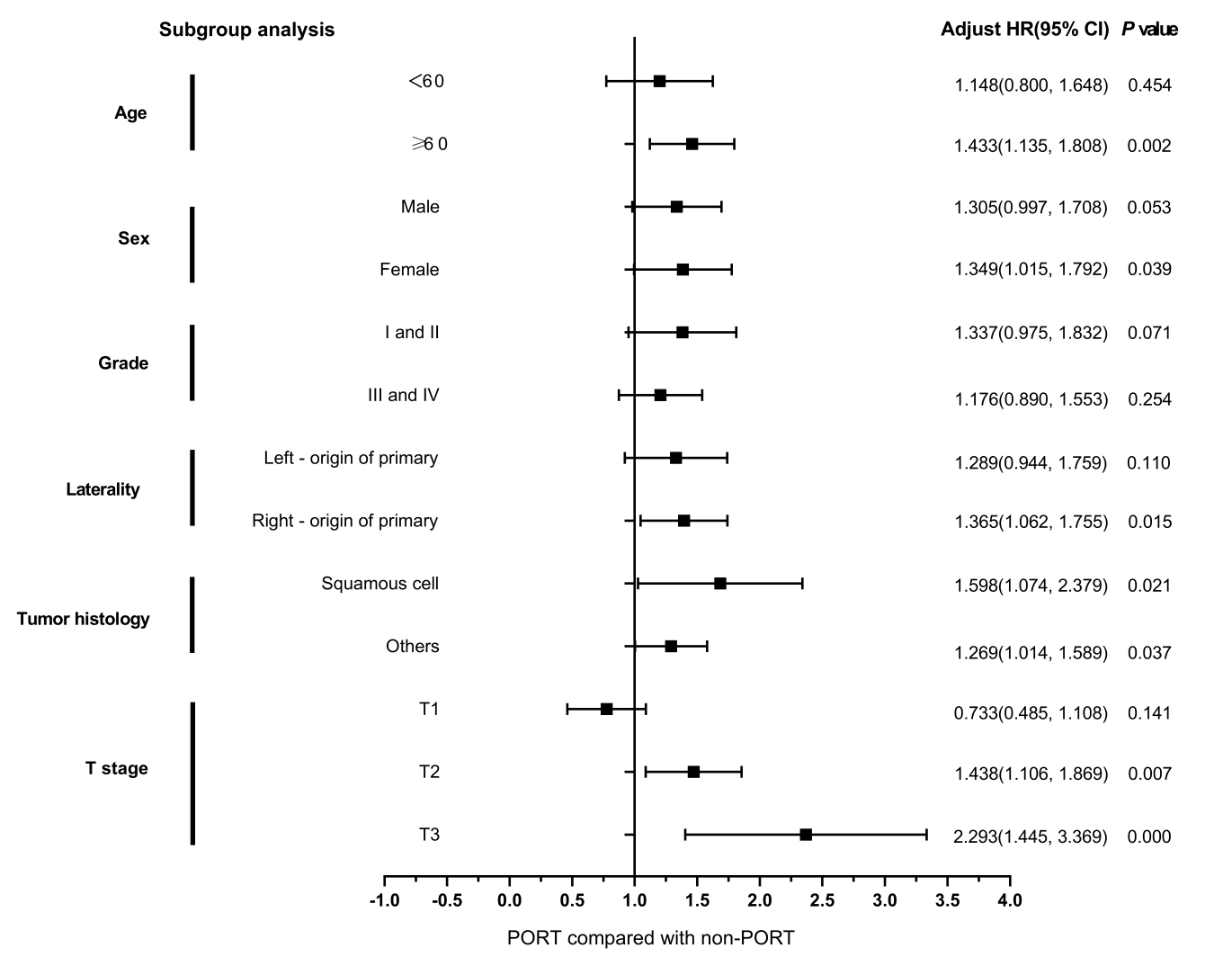

Supplement: Supplementary Figure 2 — Forest plots displaying the relationship between the PORT group and the non-PORT within the different subgroups. [file Image_2.tif]
