# Supplementary material for: The prognostic value of postoperative radiotherapy in right tumor for lung related death: based on SEER database and real-world data
Source: Front Oncol. 2023 Apr 6;13:1178064. doi: 10.3389/fonc.2023.1178064 (PMC10117832; doi:10.3389/fonc.2023.1178064)
Supplement: Supplementary file 3 [file Table_1.docx]

**Supplementary Table 1**: Baseline Characteristics of Patients with ⅢA-N2 NSCLC in left laterality of OS.
