# Supplementary material for: The prognostic value of postoperative radiotherapy in right tumor for lung related death: based on SEER database and real-world data
Source: Front Oncol. 2023 Apr 6;13:1178064. doi: 10.3389/fonc.2023.1178064 (PMC10117832; doi:10.3389/fonc.2023.1178064)
Supplement: Supplementary file 4 [file Table_2.docx]

**Table 2**: Multivariate Analysis of Overall Survival for Patients with IIIA-N2 NSCLC.
